# Applications of Artificial Intelligence in Thalassemia: A Comprehensive Review

**DOI:** 10.3390/diagnostics13091551

**Published:** 2023-04-26

**Authors:** Khaled Ferih, Basel Elsayed, Amgad M. Elshoeibi, Ahmed A. Elsabagh, Mohamed Elhadary, Ashraf Soliman, Mohammed Abdalgayoom, Mohamed Yassin

**Affiliations:** 1 College of Medicine, QU Health, Qatar University, Doha P.O. Box 2713, Qatar; 2 Hematology Section, Pediatrics Department, Hamad Medical Corporation (HMC), Doha P.O. Box 3050, Qatar; 3 Hematology Section, Medical Oncology, National Center for Cancer Care and Research (NCCCR), Hamad Medical Corporation (HMC), Doha P.O. Box 3050, Qatar

**Keywords:** artificial intelligence, thalassemia, diagnosis, B-thalassemia, iron deficiency anemia

## Abstract

Thalassemia is an autosomal recessive genetic disorder that affects the beta or alpha subunits of the hemoglobin structure. Thalassemia is classified as a hypochromic microcytic anemia and a definitive diagnosis of thalassemia is made by genetic testing of the alpha and beta genes. Thalassemia carries similar features to the other diseases that lead to microcytic hypochromic anemia, particularly iron deficiency anemia (IDA). Therefore, distinguishing between thalassemia and other causes of microcytic anemia is important to help in the treatment of the patients. Different indices and algorithms are used based on the complete blood count (CBC) parameters to diagnose thalassemia. In this article, we review how effective artificial intelligence is in aiding in the diagnosis and classification of thalassemia.

## 1. Introduction

Thalassemia is an autosomal recessive inherited genetic disorder, which is classified as hypochromic microcytic anemia [1]. The degrees of hypochromic microcytic anemia in thalassemia vary depending on the genetic defects of alpha-globulin or beta-globulin genes [2]. It was found that 5% of the world’s population has a globin variant with 1.7% with alpha-thalassemia or beta-thalassemia trait. Men and women are affected equally by thalassemia [3,4]. Thalassemia is predominant in the Mediterranean, African, and Southeast Asian populations [5,6]. The hemoglobin structure is made normally of two alpha and two beta chains [7]. Thalassemia is characterized by a defect in the alpha chain or the beta chain, which will lead to the alpha-thalassemia and beta-thalassemia diseases, respectively [8]. B-thalassemia is the most common type of thalassemia diseases [9]. Moreover, B-thalassemia is classified according to the severity into thalassemia major, thalassemia intermedia, thalassemia minor, and silent thalassemia [10]. Patients with thalassemia minor are usually asymptomatic and do not require treatment. Most patients with thalassemia minor are diagnosed incidentally when their blood test shows a mild hypochromic microcytic anemia. There are many causes of hypochromic microcytic anemia, mainly iron deficiency and thalassemia and sideroblastic anemia. Other parameters are used, such as the mean corpuscular volume (MCV) and the red blood cell distribution width (RDW), in addition to the patient’s history to exclude the other causes of thalassemia [11,12]. The RDW is the most important parameter used to differentiate between iron deficiency anemia and thalassemia. The RDW value is higher than the normal value in 90% of patients with iron deficiency, but in only 50% of patients with thalassemia [13,14]. Additional investigations required the establishment of the diagnosis including serum ferritin, peripheral smear, hemoglobin electrophoresis, and serum lead level [12]. However, the diagnosis of thalassemia subtypes can be established only using the genetic analysis for the α- and β-globin genes [12].

Thalassemia major is the most severe form of thalassemia and it is characterized by severe anemia and skeletal deformities, which requires blood transfusions for survival [15]. However, long-term blood transfusion has many complications on the different body systems due to the depositions of iron in different organs. Iron overload affects all the body organs, particularly the heart, liver, and the endocrine system organs. Therefore, transfusion-dependent patients will develop iron overload and require chelation therapy to remove the excess iron and decrease the complications of iron overload [16,17,18]. 

To date, there are three major classes of iron chelators: Hexadentate (deferoxamine [DFO], Desferal^®^, Novartis Pharma AG, Basel, Switzerland), in which one atom of iron is bound to one DFO molecule; bidentate (deferiprone, [DFP] Ferriprox^®^, Apotex Inc., Toronto, ON, Canada), in which one atom of iron is bound to three DFP molecules; and tridentate (deferasirox [DFX], Exjade^®^ and Jadenu^®^, Novartis Pharma AG, Basel, Switzerland), in which one atom of iron is bound to two DFX molecules [19]. The intensive demands and uncomfortable side effects of therapy can have a negative impact on daily activities and well-being, which may affect adherence to treatment [20]. The new tablet DFX formulation (Jadenu^®^) was developed to overcome these tolerability issues and is the only once-daily oral iron chelator that can be swallowed with a light meal, without the need to disperse into a suspension prior to consumption [21]. However, it was found that short-term treatment with Jadenu^®^ is safe, but is associated with a non-significant decrease in LIC and serum ferritin levels [21].

There are many complications of thalassemia. One of the complications is the decrease in fertility in men by affecting the sperms’ motility, count, and morphology [22]. A recent study showed a significant increase in the levels of luteinizing hormone (LH) and follicle-stimulating hormone (FSH), in addition to the count and motility of the sperms 7 days after the initiation of blood transfusion therapy in thalassemic patients. Moreover, blood transfusion plays a critical role in decreasing the risk of osteoporosis in thalassemic patients by increasing the insulin-like growth factor-1 (IGF) and IGF-binding protein-3 (BP) secretion [23,24]. This is due to the fact that the IGF-1 is a key hormone and plays a major role in cell growth and protein turnover, acting as the primary mediator of many of the responses regulated by GH in tissues [25]. In addition, a recent study showed that a denosumab therapy for 1 year is associated with increased bone mineral density (BMD) at the lumbar spine (LS) and femoral neck (FN) of patients with beta-thalassemia major (BTM) and was associated with a rapid and sustained reduction in type 1 collagen carboxy telopeptide (ICCT) levels [26]. The thyroid gland is commonly affected in thalassemic patients. Primary hypothyroidism is the most common disease, and it occurs due to abnormalities of the thyroid gland. However, central hypothyroidism (CH) has been reported as well in thalassemic patients due to damage to the pituitary gland, but it is less common than primary hypothyroidism. The diagnosis of this condition is usually made on a biochemical basis showing low circulating concentrations of thyroid hormone associated with an inappropriately low TSH level [27]. Furthermore, insulin hormone resistance is one of the complications in thalassemia, and the use of continuous glucose monitoring system (CGMS) is the most reliable method in the diagnosis of insulin resistance and hyperglycemia in thalassemic patients [28]. Additionally, progressive GFR decline through the typical pathway of hyperfiltration—albuminuria—progressive renal injury, leading to the progression of chronic kidney disease (CKD) [29,30]. A recent study showed that the renal stress test (RST) can be used in a β-TM population to evaluate the decline in the renal function and correlate it to the iron overload, as well as to provide a guide parameter in assessing patients’ renal injury and their susceptibility to AKI [31]. There are multiple indices which are derived from the complete blood count parameters to differentiate between thalassemia and other diseases, which include the hemoglobin (Hb), mean corpuscular hemoglobin (MCH), mean corpuscular hemoglobin concentration (MCHC), mean corpuscular volume (MCV), and the red blood cell distribution width (RDW).

MCV evaluation is a useful tool for screening for α-thalassemia-1 and β-thalassemia traits during pregnancy due to its simplicity, low cost, and high sensitivity. Positive MCV tests (≤80° fl) showed a sensitivity of 92.9% and specificity of 83.9% in screening for α-thalassemia-1 and β-thalassemia traits, respectively. The positive predictive value and negative predictive value were 37.9% and 99.1%, respectively. Examples of these indices include Shine and Lal (S&L), Ricerca, Green and King (G&K), RDW index, and mean cell hemoglobin density (MCHD) index [14,32,33,34,35,36]. Among these indices, the Green-King index and RDW index are the most commonly used due to their high sensitivity and specificity [32]. Therefore, artificial intelligence can play a crucial role in diagnosing and differentiating thalassemia from other diseases, particularly iron deficiency anemia, which is important for guiding the management of the patients [37,38].

## 2. Materials and Methods

### 2.1. Data Sources and Search Strategy

Our search strategy was developed using PubMed’s Medical Subject Headings (MeSH) terms, along with other title and abstract keywords. For our disease of interest (thalassemia), we included terms related to thalassemia diseases and its subtypes, such as “B-thalassemia”, “A-thalassemia”, “Thalassemia”, and other terms of this nature to avoid missing any related articles. To include articles discussing the use of AI in thalassemia, we also included terms for machine learning (ML), such as “artificial intelligence”, “machine-learning”, “AI”, and “indices”. All the studies identified by the search strategy were moved into EndNote where duplicates were removed. A total number of 28 studies were found in the database. Out of the 28, 16 studies were included in the review.

### 2.2. Inclusion Criteria

This review included original research articles that discuss the use of ML algorithms in the various types of thalassemia in humans. Full text articles that submitted abstracts and conference abstracts were all included within our study. Studies were excluded from our study for the following reasons: (1) Animal studies, (2) reviews or non-original articles, (3) non-English articles. 

### 2.3. Data Collection and Extraction

The data that have been collected in this paper include the type of study, publication year, outcome assessed, model utilized, mode evaluation (sensitivity, specificity, accuracy, area under the curve), and strengths and limitations. 

## 3. Results

### 3.1. Artificial Intelligence in Diagnosing Thalassemia

It is crucial to differentiate between thalassemia and other causes of microcytic anemia to decrease the number of investigations needed and help the physicians focus on the clinical evaluation and management of non-thalassemia diseases by skipping unnecessary tests. As a result, the importance of AI in diagnosing thalassemia is increasing and different indices and web-based prediction tools are developed to aid in the diagnosis of thalassemia and help in reducing the resources and investigations ordered by the physicians to distinguish between thalassemia and the other non-thalassemia causes of microcytic anemia. 

**Figure 1:**
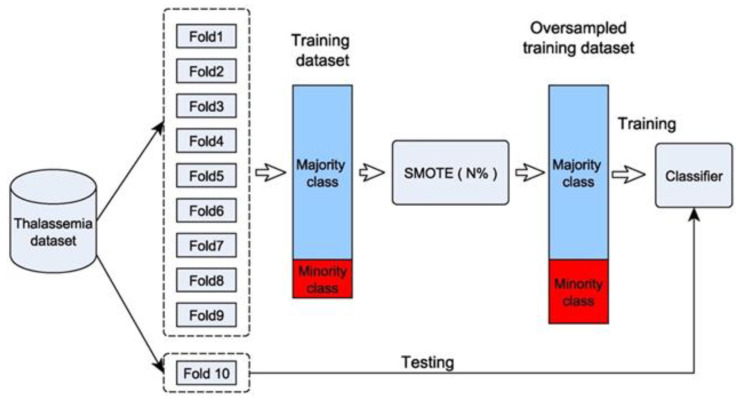
Illustration of the cross-validation scheme with the SMOTE oversampling applied only on the training data.

There are different machine learning algorithms that are used to diagnose thalassemia using complete blood count (CBC) parameters with high sensitivity and specificity values. A model proposed by AlAgha et al. used the k-nearest neighbor (k-NN), Naïve Bayesian (NB), decision tree (DT), and multi-layer perceptron (MLP) neural network classification algorithms. The k-NN is a classification algorithm which works by classifying unknown instances based on the nearest known instances. The main idea of this algorithm is that instances that are located close to each other are likely to belong to the same class. However, instances that are distant from each other are less likely to belong to the same class. The MLP, which is a type of artificial neural network (ANN), works by distributing neurons over several layers, which are an input layer, an output layer, and one or more hidden layers. In MLP, neurons in the input layer receive the data, and then the neurons in the hidden layer process and send them to the output layer. For the decision process, a mathematical function called the “activation function” is defined. The Naïve Bayesian classifier adopts the idea that the effect of a certain feature on a given class is independent to the existence of any other feature, which is designed based on Bayes’ theorem. Decision trees are machine-learning models represented as tree structures, where each internal node is a condition on a selected feature and each branch of the node is a result of its condition. In this method, to overcome the problem of the highly imbalanced class distribution in the dataset, a balancing technique called SMOTE is proposed and applied to handle this problem. Figure 1 shows the use of the SMOTE technique on the training data. This model successfully reached a sensitivity and specificity of 98.4% and 98.7%, respectively [39]

**Figure 2:**
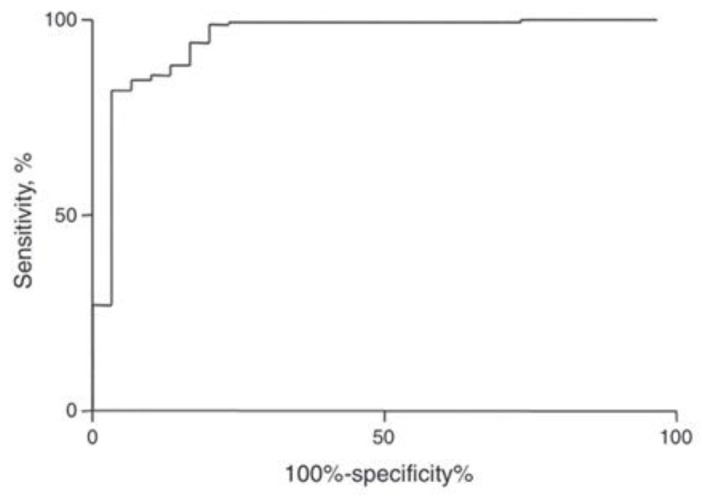
The receiver operating characteristic curve for the Matos and Carvalho index.

A new index was established based on a mathematical formula to discriminate between iron deficiency anemia (IDA) and thalassemia trait (TT). The used values of the hematological parameters of 23 patients with TT and 83 patients with IDA were confirmed using gold standard tests. The gold standard test for diagnosing TT is an HbA2 level of more than 3.5% and the gold standard is low serum ferritin in IDA. The adjusted formula of the new index developed in this study is Matos and Carvalho Index (MCI) = (1.91 × RBC) + (0.44 × MCHC). According to the ROC curve, the MCI presented a cut-off point with a value of 23.85 to discriminate between IDA and TT. If the index is <23.85, the patient is classified as an IDA patient, while values >23.85 classify the individual as a TT carrier. Moreover, TT simple decision tree based on indices were used in the diagnosis of thalassemia. To validate the new index, a new cross-sectional study was conducted from 2009 to 2011 in two different hospitals and a total of 227 outpatients were included in the study. Figure 2 shows the receiver operating characteristic curve for the Matos and Carvalho index. The sensitivity of the index is 99.3% and specificity is 76.6%, respectively [40]. 

**Figure 3:**
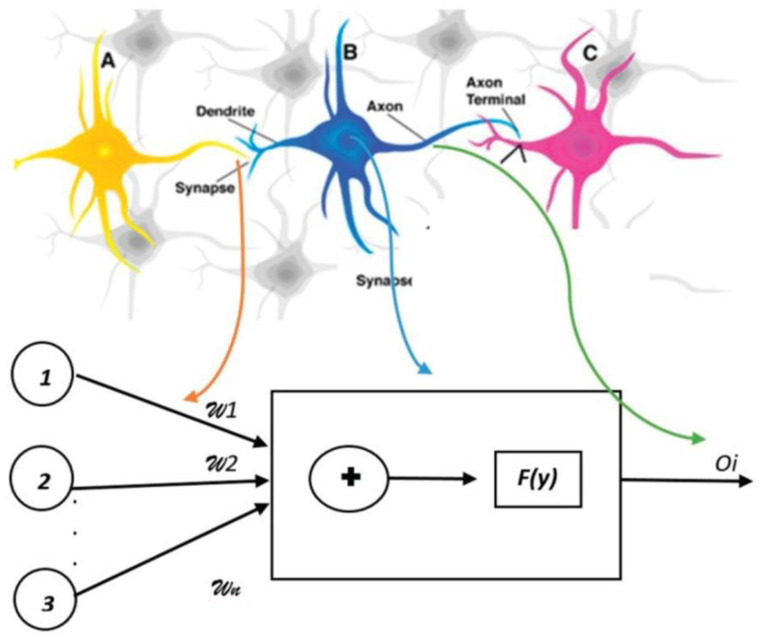
Comparison of artificial neural networks (ANN) and biological neurons.

**Figure 4:**
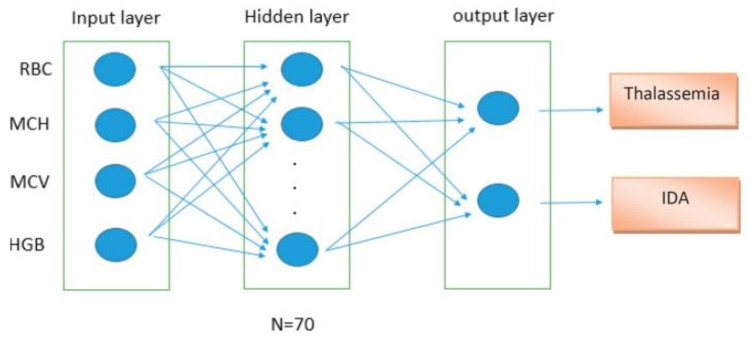
The architecture of the proposed artificial neural network (ANN).

A neural network-based model was proposed for the accurate and timely identification of IDA and β-thalassemia discrimination. Artificial neural network (ANN) is an effective screening method for the initial diagnosis and management of diseases. The algorithm of the ANN is based on the processing of experimental data (input), and transfers the knowledge or logical data model (hidden layer) into a network structure. Figure 3 shows a comparison of artificial neural networks (ANN) and biological neurons. In the study, the information of 268 patients was selected and divided into two groups of IDA and thalassemia. The random sampling technique was used regardless of the sex and age criteria. All patients with thalassemia had HbA2 levels above 3.5% and iron deficiency was confirmed by the ferritin test (<8.0 ng/mL) in women and (<28.0 ng/mL) in men. The architecture of the proposed ANN is shown in Figure 4. The model showed sensitivity of 93.13% and specificity of 92.33%, respectively [41].

Moreover, algorithms based on the principal component analysis (PCA) were used to diagnose thalassemia. The data used in this model are composed of six which are sex, age, Hb, Hct, RBC, and RDW. Specifically, Hb is hemoglobin, Hct is hematocrit, RBC is the red blood cell count, and RDW is the red cell distribution width. Then, the data were analyzed to know the possible impact of each parameter on the classification. Principle component analysis (PCA) along with two- and three-dimensional plots of the dataspace were used for this purpose. In order to rank the impact of the parameters and determine which parameters to plot in the analysis, five feature selection methods were used and the results are presented in Table 1. In all cases, RBC is found to be the most important feature, with RDW identified as the second most important four out of five times. Gender is removed by the feature selection methods and ranked last by the methods that rank features. The results show that machine-learning classifiers produce good overall accuracy and are able to identify instances of the co-occurrence class in contrast to the existing methods [42].

**Figure 5:**
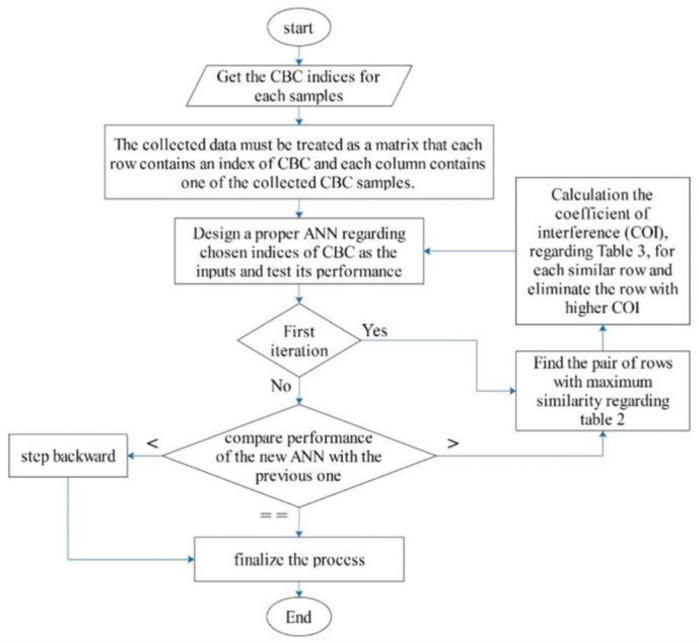
The proposed architecture naming pattern-based input selection artificial neural network (PBIS-ANN).

However, pattern-based input selection artificial neural network (PBIS-ANN) architecture was used for the diagnosis of thalassemia. A brief illustration of the proposed method has been shown in Figure 5. The dataset contains 532 CBC samples from IDA and β-TT subjects. The results of the new mode showed higher efficacy compared to some of the popular traditional methods, such as Mentzer index (MI) and the multilayer perceptron (MLP). Sensitivity (SENS), specificity (SPEC), positive predictive value (PPV), negative predictive value (NPV), accuracy (ACC), and Youden’s index (YI) were used for comparison [43].

An article published by Laengsri et al. in 2019 discussed a new web-based prediction tool called ThalPred for discriminating thalassemia trait from iron deficiency anemia in Thailand. The study collected the data retrospectively from patients with hypochromic microcytic anemia from different health centers in Thailand between the period from July 2014 to September 2016. They used five machine-learning models, including k-nearest neighbor (k-NN), random forest (RF), and artificial neural network (ANN) to construct a discriminant model. Thereafter, the performance was assessed and compared with multiple other existing indices. The authors were able to establish a web-based tool, by which users can easily achieve their desired screening test result without the need to carry out the underlying mathematical and computational details, and they achieved prediction results with an external accuracy, MCC, and AUC of 95.59, 0.87, and 0.98, respectively [44].

Another study by Yi-Kai Fu et al., which was performed in Taiwan to establish an index for the diagnosis of thalassemia using a total of 350 Taiwanese adult patients, retrospectively reviewed their anemia diagnosis, complete blood cell counts, and hemoglobin gene profiles. Then, the authors applied multiple existing indices on their cohorts, and compared the sensitivity, specificity, positive, and negative predictive values. The new index established by the author showed an average AUC of 0.76 and average error rate of 0.26, which surpassed all other indices. However, their approach needs to be validated in other studies or a larger database [45].

A paper published by Golosio et al. compared two techniques that used artificial intelligence and pattern recognition in the diagnosis and classification of thalassemia patients, which are the support vector machine (SVM) and the k-nearest neighbor (k-NN). These techniques were used to distinguish between thalassemia and non-thalassemia, and then among the thalassemia patients, the authors classified patients into alpha-thalassemia cases or beta-thalassemia cases. Sensitivity and specificity were used to compare the two methods.

Both techniques showed high specificity that reached 95% in differentiating between thalassemia patients and healthy subjects. However, the sensitivity of MLP is 92% compared to 83% in the SVM method [46]. Another article discussed how the neural network can be used in diagnosing thalassemia. The method is evolved by genetic programming (GP), which used the values of red blood cells, reticulocytes, and other blood test parameters [47].

An article showed that the classification methods using machine-learning techniques mostly depend on the quality of the data used in the learning process. In the paper, they discussed how the data of beta-thalassemia are used to develop a method for screening thalassemia using the MCH, MCV, and hemoglobin values [48]. It was proven by another study that machine learning can be used by physicians to aid in the screening and diagnosis of thalassemia and other Hb variants, which is carried out using different classifiers. For instance, the decision tree and random forest classifiers were proven to be the main methods in terms of classifying the Hb variants. Moreover, studying a large number of samples may help in generating greater precision and F1-score values, which imply greater accuracy [49]. Moreover, two predictive methods were established, one method for the detection of B-thalassemia trait (BTT), and the other for the identification of hemoglobin E (HbE) trait and BTT using the decision tree, Naïve Bayesian classifier, and artificial neural network. After validation of the scores, the scores showed sensitivity of 100% of both the scores with their respective threshold values. The results revealed the specificity of the screening scores to be 79.25% and 91.74% for BTT and 58.62% and 78.03% for the joint score of HbE and BTT, respectively. This index showed high sensitivity and specificity compared to Youden’s index. As a result, the proposed scores can obviate a large portion of the population from expensive high-performance liquid chromatography (HPLC) analysis during the screening of BTT, and joint determination of BTT and HbE, respectively, thereby saving significant resources and cost that are currently being utilized for screening purposes. Table 2 shows a comparison of some of the existing indices using the proposed method [50].

Additionally, a decision support system to distinguish between β-thalassemia and iron deficiency anemia was developed. Logistic regression, k-nearest neighbor, the support vector machine, extreme learning machine, and regularized extreme learning machine classification algorithms were used in the proposed system (Table 3). Classification performance was evaluated using accuracy, sensitivity, F-measure, and specificity parameters obtained from 342 patients and showed 96.30% accuracy for female gender and 94.37% for male gender [53]. A new method that uses a new technique called feature fusion was developed to classify the patients with hypochromic microcytic anemia and know whether the cause of the anemia is due to thalassemia or other causes. Feature fusion indicates the use of blood smear image features, which are extracted by the deep convolutional neural network (CNN), in addition to the clinical values of the complete blood tests. This combination will be used to have a better prediction of the cause of the microcytic hypochromic anemia. K-nearest neighbor (k-NN), support vector machine (SVM), and neural network classification models were used in this study to predict the causes of the microcytic hypochromic anemia. The method was applied, and the feed-forward-back-propagation neural network achieved 99% accuracy, 100% sensitivity, and 98% specificity, respectively [54].

### 3.2. Artificial Intelligence (AI) and the Complications of Thalassemia

One of the major complications of thalassemia is iron overload, which results from blood transfusions. AI is now being used in measuring the amount of iron in the organs to help in the prevention and treatment of thalassemia complications. There are many limitations to measure the iron concentration in different organs of the body. For instance, in the liver, this is due to the limitations in measuring serum ferritin and performing liver biopsy to know the iron liver iron concentration (LIC). A recent study was carried out to evaluate the performance of an automated deep-learning-based medical device (DLA R2-MRI) for measuring liver iron concentration from MRI using an independent multi-scanner dataset. They used 1395 datasets from 66 different scanners submitted for expert manual analysis using spin-density projection assisted (SDPA) R2-MRI (the reference standard). The sensitivities and specificities of the automated system for predicting LIC values were >90% [55]. MRI approach by estimation of the liver T2* value is the most established evaluation technique for liver iron. A recent retrospective study aimed to develop a new deep-learning method for an evaluation of the iron content in the liver using the T2* value. A total of 1069 thalassemia major patients enrolled in the core laboratory of the Myocardial Iron Overload in Thalassemia (MIOT) network, which were included in the training (80%) and test (20%) sets. Four deep-learning convolutional neural networks (HippoNet-2D, HippoNet-3D, HippoNet-LSTM, and an ensemble network HippoNet-Ensemble) were used to achieve unsupervised staging of LIC using five classes (normal, borderline, middle, moderate, severe). The training set was employed to construct the deep-learning model. The performance of the LIC staging model was evaluated in the test set and in the external test set. The model’s performances were assessed by evaluating the accuracy, sensitivity, and specificity with respect to the ground truth labels obtained by T2* measurements and by comparison with operator-induced variability originating from different regions of interest (ROI) placements. The results showed that the HippoNet-Ensemble reached an accuracy significantly higher than the other networks, and a sensitivity and specificity higher than HippoNet-LSTM. This indicates that the proposed HippoNet-Ensemble network can perform unsupervised LIC staging and achieves a good prognostic performance [56].

## 4. Conclusions

In the above-mentioned papers, it has been shown that machine-learning techniques play a crucial role in the diagnosis of thalassemia disease. This review describes the effective parameters for the diagnosis of thalassemia based on the CBC test. The classification performance of KNN, MLP, NN, decision tree, and support vector machine were evaluated. The results from the papers have shown that the neural network is the best method for diagnosing thalassemia and other blood diseases using parameters, such as MCH, MCHC, and RDW. Additionally, the most effective factors for diagnosing thalassemia are RBC, HGB, MCV, and Hematocrit, which help in diagnosing the disease in a person through an examination of these parameters. Moreover, the studies showed that the signed sigmoid was the best activation function in the input layer. Furthermore, early diagnosis of thalassemia helps in deciding on its treatment and preventing the severity of the disease and its consequences.

## Figures and Tables

**Table 1:** The feature ranking order.

Method	Feature Ranking Order
cfsSubsetEval	RBC, RDW
Attribute Correlation	RBC, age, RDW. Hb, Hct
Gain Ratio	RBC. RDW. Hb. age, Hct, gender
Symmetrical Uncertainty	RBC. RDW. Hb, age, Hct, gender
Information Gain	RBC. RDW. Hb. age, Hct, gender

**Table 2:** Comparative outcomes of proposed scoring mechanisms with existing indices that are used to diagnose the thalassemia disease.

Index	Formula	BTT	Sensitivity	Specificity	PPV	NPV	Efficiency	Youden’s Index
Mentzer [11]	MCVRBC	<13	70.31	96.28	86.54	90.50	89.68	66.59
Srivastava [51]	MCHRBC	<3.8	62.50	97.34	88.89	88.40	88.49	59.84
Shine and Lal [52]	MCV2 × MCH100	<1530	95.31	79.79	61.62	98.04	83.73	75.10
Jayabose, et al. [33]	MCV × RDWRBC	<220	64.06	90.96	70.69	88.14	84.13	55.02
Sirdah, et al. [32]	MCV − RBC − 3Hb	<27	64.06	97.34	89.13	88.83	88.89	61.40
Ehsani, et al. [35]	MCV − 10RBC	<15	68.75	96.81	88	90.10	89.68	65.56

BTT: Beta-thalassemia trait; PPV: Positive predictive value; NPV: Negative predictive value.

**Table 3:** Performance metrics and outcomes for the best models used in the diagnosis of thalassemia disease in the included full-text articles.

Study	Outcomes	AUC	ACC	SE	SP
AlAgha, et al. [39]	Identification of thalassemia	99.8%	99.8%	99.5%	99.5%
Matos, et al. [40]	Identification of thalassemia	95.7%	95%	76.7%	99.3%
Kabootarizadeh L., et al. [41]	Identification of thalassemia	--	92.5%	93.1%	92.3%
Jamei, M.K. [43]	Identification of thalassemia	--	98%	97.7%	98.4%
Purwar, S., et al. [52]	Identification of thalassemia	--	99%	100%	98%
Ullah, Z., et al. [44]	Identification of thalassemia	--	--	100%	93%

AUC: Area under the curve; ACC: Accuracy; SE: Sensitivity; SP: Specificity.

## Data Availability

Not applicable.

## References

[1] Munkongdee T., Chen P., Winichagoon P., Fucharoen S., Paiboonsukwong K. (2020). Update in Laboratory Diagnosis of Thalassemia. Front. Mol. Biosci..

[2] De Sanctis V., Kattamis C., Canatan D., Soliman A.T., Elsedfy H., Karimi M., Daar S., Wali Y., Yassin M., Soliman N. (2017). β-Thalassemia Distribution in the Old World: An Ancient Disease Seen from a Historical Standpoint. Mediterr. J. Hematol. Infect. Dis..

[3] Angastiniotis M., Modell B. (1998). Global epidemiology of hemoglobin disorders. Ann. N. Y. Acad. Sci..

[4] Rund D., Rachmilewitz E. (2005). β-Thalassemia. N. Engl. J. Med..

[5] Kassebaum N.J., Jasrasaria R., Naghavi M., Wulf S.K., Johns N., Lozano R., Regan M., Weatherall D., Chou D.P., Eisele T.P. (2014). A systematic analysis of global anemia burden from 1990 to 2010. Blood.

[6] Frangoul H., Altshuler D., Cappellini M.D., Chen Y.-S., Domm J., Eustace B.K., Foell J., de la Fuente J., Grupp S., Handgretinger R. (2020). CRISPR-Cas9 Gene Editing for Sickle Cell Disease and β-Thalassemia. N. Engl. J. Med..

[7] Lal A., Wong T.E., Andrews J., Balasa V.V., Chung J.H., Forester C.M., Ikeda A.K., Keel S.B., Pagano M.B., Puthenveetil G. (2018). Transfusion practices and complications in thalassemia. Transfusion.

[8] Viprakasit V., Ekwattanakit S. (2018). Clinical Classification, Screening and Diagnosis for Thalassemia. Hematol. Oncol. Clin. N. Am..

[9] Ayyıldız H., Tuncer S.A. (2020). Determination of the effect of red blood cell parameters in the discrimination of iron deficiency anemia and beta thalassemia via Neighborhood Component Analysis Feature Selection-Based machine learning. Chemom. Intell. Lab. Syst..

[10] Khan A.M., Al-Sulaiti A.M., Younes S., Yassin M., Zayed H. (2021). The spectrum of beta-thalassemia mutations in the 22 Arab countries: A systematic review. Expert Rev. Hematol..

[11] Mentzer W.C. (1973). Differentiation of iron deficiency from thalassaemia trait. Lancet.

[12] Brancaleoni V., Di Pierro E., Motta I., Cappellini M.D. (2016). Laboratory diagnosis of thalassemia. Int. J. Lab. Hematol..

[13] Flynn M.M., Reppun T.S., Bhagavan N.V. (1986). Limitations of red blood cell distribution width (RDW) in evaluation of microcytosis. Am. J. Clin. Pathol..

[14] England J.M., Fraser P.M. (1973). Differentiation of iron deficiency from thalassaemia trait by routine blood-count. Lancet.

[15] Yassin M.A., Soliman A.T., De Sanctis V., Yassin K.S., Abdulla M.A. (2019). Final Height and Endocrine Complications in Patients with β-Thalassemia Intermedia: Our Experience in Non-Transfused Versus Infrequently Transfused Patients and Correlations with Liver Iron Content. Mediterr. J. Hematol. Infect. Dis..

[16] Maggio A., Vitrano A., Capra M., Cuccia L., Gagliardotto F., Filosa A., Magnano C., Rizzo M., Caruso V., Gerardi C. (2009). Improving survival with deferiprone treatment in patients with thalassemia major: A prospective multicenter randomised clinical trial under the auspices of the Italian Society for Thalassemia and Hemoglobinopathies. Blood Cells Mol. Dis..

[17] Telfer P., Coen P.G., Christou S., Hadjigavriel M., Kolnakou A., Pangalou E., Pavlides N., Psiloines M., Simamonian K., Skordos G. (2006). Survival of medically treated thalassemia patients in Cyprus. Trends and risk factors over the period 1980–2004. Haematologica.

[18] Farmaki K., Tzoumari I., Pappa C., Chouliaras G., Berdoukas V. (2010). Normalisation of total body iron load with very intensive combined chelation reverses cardiac and endocrine complications of thalassaemia major. Br. J. Haematol..

[19] Neufeld E.J. (2006). Oral chelators deferasirox and deferiprone for transfusional iron overload in thalassemia major: New data, new questions. Blood.

[20] Fortin P.M., Fisher S.A., Madgwick K.V., Trivella M., Hopewell S., Doree C., Estcourt L.J. (2018). Interventions for improving adherence to iron chelation therapy in people with sickle cell disease or thalassaemia. Cochrane Database Syst. Rev..

[21] Yassin M.A., Soliman A.T., De Sanctis V., Hussein R.M., Al-Okka R., Kassem N., Ghasoub R., Basha A., Nashwan A.J., Adel A.M. (2018). Jadenu(^®^) Substituting Exjade(^®^) in Iron Overloaded β-Thalassemia Major (BTM) Patients: A Preliminary Report of the Effects on the Tolerability, Serum Ferritin Level, Liver Iron Concentration and Biochemical Profiles. Mediterr. J. Hematol. Infect. Dis..

[22] Soliman A., Yasin M., El-Awwa A., Osman M., de Sanctis V. (2012). Acute effects of blood transfusion on pituitary gonadal axis and sperm parameters in adolescents and young men with thalassemia major: A pilot study. Fertil. Steril..

[23] Soliman A.T., Abushahin A., Abohezeima K., Khalafallah H., Adel A., Elawwa A., Elmulla N. (2011). Age related IGF-I changes and IGF-I generation in thalassemia major. Pediatr. Endocrinol. Rev..

[24] A Yassin M., Soliman A.T., De Sanctis V., Abdula M.A., Riaz L.M., Ghori F.F., Yousaf A., Nashwan A.J., Abusamaan S., Moustafa A. (2018). Statural Growth and Prevalence of Endocrinopathies in Relation to Liver Iron Content (LIC) in Adult Patients with Beta Thalassemia Major (BTM) and Sickle Cell Disease (SCD). Acta Biomed. Atenei Parm..

[25] De Sanctis V., Soliman A.T., Candini G., Yassin M., Raiola G., Galati M.C., Elalaily R., Elsedfy H., Skordis N., Garofalo P. (2014). Insulin-like Growth Factor-1 (IGF-1): Demographic, Clinical and Laboratory Data in 120 Consecutive Adult Patients with Thalassaemia Major. Mediterr. J. Hematol. Infect. Dis..

[26] Yassin M.A., Soliman A.T., De Sanctis V., Abdelrahman M.O., Aziz Bedair E.M., AbdelGawad M. (2014). Effects of the anti-receptor activator of nuclear factor kappa B ligand denusomab on beta thalassemia major-induced osteoporosis. Indian J. Endocrinol. Metab..

[27] De Sanctis V., Soliman A., Candini G., Campisi S., Anastasi S., Iassin M. (2013). High prevalence of central hypothyroidism in adult patients with β-thalassemia major. Georgian Med. News.

[28] Soliman A.T., Yasin M., El-Awwa A., De Sanctis V. (2013). Detection of glycemic abnormalities in adolescents with beta thalassemia using continuous glucose monitoring and oral glucose tolerance in adolescents and young adults with β-thalassemia major: Pilot study. Indian J. Endocrinol. Metab..

[29] Ravarotto V., Simioni F., Pagnin E., Davis P.A., Calò L.A. (2018). Oxidative stress–chronic kidney disease–cardiovascular disease: A vicious circle. Life Sci..

[30] Ravarotto V., Bertoldi G., Innico G., Gobbi L., Calò L.A. (2021). The Pivotal Role of Oxidative Stress in the Pathophysiology of Cardiovascular-Renal Remodeling in Kidney Disease. Antioxidants.

[31] Nalesso F., Rigato M., Cirella I., Protti M.P., Zanella R., Rossi B., Putti M.C., Martino F.K., Calò L.A. (2022). The Assessment of Renal Functional Reserve in β-Thalassemia Major Patients by an Innovative Ultrasound and Doppler Technique: A Pilot Study. J. Clin. Med..

[32] Sirdah M., Tarazi I., Al Najjar E., Al Haddad R. (2008). Evaluation of the diagnostic reliability of different RBC indices and formulas in the differentiation of the β-thalassaemia minor from iron deficiency in Palestinian population. Int. J. Lab. Hematol..

[33] Jayabose S., Giamelli J., LevondogluTugal O., Sandoval C., Ozkaynak F., Visintainer P. (1999). #262 Differentiating iron deficiency anemia from thalassemia minor by using an RDW-based index. J. Pediatr. Hematol. Oncol..

[34] Huber A., Ottiger C., Risch L., Regenass S., Hergersberg M., Herklotz R. (2004). Thalassämie-Syndrome: Klinik und Diagnose. Swiss Medical Forum.

[35] Green R., King R. (1989). A new red cell discriminant incorporating volume dispersion for differentiating iron deficiency anemia from thalassemia minor. Blood Cells.

[36] Ehsani M., Shahgholi E., Rahiminejad M., Seighali F., Rashidi A. (2009). A new index for discrimination between iron deficiency anemia and beta-thalassemia minor: Results in 284 patients. Pak. J. Biol. Sci. PJBS.

[37] Camaschella C. (2015). Iron-Deficiency Anemia. N. Engl. J. Med..

[38] Killip S., Bennett J.M., Chambers M.D. (2007). Iron deficiency anemia. Am. Fam. Physician.

[39] AlAgha A.S., Faris H., Hammo B.H., Al-Zoubi A.M. (2018). Identifying β-thalassemia carriers using a data mining approach: The case of the Gaza Strip, Palestine. Artif. Intell. Med..

[40] Matos J.F., Dusse L.M.S., Borges K.B.G., de Castro R.L.V., Coura-Vital W., Carvalho M.d.G. (2016). A new index to discriminate between iron deficiency anemia and thalassemia trait. Rev. Bras. Hematol. Hemoter..

[41] Kabootarizadeh L., Jamshidnezhad A., Koohmareh Z. (2019). Differential Diagnosis of Iron-Deficiency Anemia from β-Thalassemia Trait Using an Intelligent Model in Comparison with Discriminant Indexes. Acta Inf. Med.

[42] Bellinger C., Amid A., Japkowicz N., Victor H. Multi-label classification of anemia patients. Proceedings of the 2015 IEEE 14th International Conference on Machine Learning and Applications (ICMLA).

[43] Jamei M.K., Talarposhti K.M. (2016). Discrimination between Iron Deficiency Anaemia (IDA) and β-Thalassemia Trait (β-TT) Based on Pattern-Based Input Selection Artificial Neural Network (PBIS- ANN). J. Adv. Comput. Res..

[44] Ullah Z., Khattak A.A., Ali S.A., Hussain J., Noor B., Bano R., Jan Mahsud M.A. (2016). Evaluation of five discriminating indexes to distinguish Beta-Thalassemia Trait from Iron Deficiency Anaemia. J. Pak. Med. Assoc..

[45] Fu Y.-K., Liu H.-M., Lee L.-H., Chen Y.-J., Chien S.-H., Lin J.-S., Chen W.-C., Cheng M.-H., Lin P.-H., Lai J.-Y. (2021). The TVGH-NYCU Thal-Classifier: Development of a Machine-Learning Classifier for Differentiating Thalassemia and Non-Thalassemia Patients. Diagnostics.

[46] Amendolia S.R., Cossu G., Ganadu M.L., Golosio B., Masala G., Mura G.M. (2003). A comparative study of K-Nearest Neighbour, Support Vector Machine and Multi-Layer Perceptron for Thalassemia screening. Chemom. Intell. Lab. Syst..

[47] Wongseree W., Chaiyaratana N., Vichittumaros K., Winichagoon P., Fucharoen S. (2007). Thalassemia classification by neural networks and genetic programming. Inf. Sci..

[48] Amin M., Habib M.A. (2015). Comparison of Different Classification Techniques Using WEKA for Hematological Data. Am. J. Eng. Res..

[49] Saikia Borah M., Bhuyan B., Pathak D., Bhattacharya P. (2018). Machine learning in predicting hemoglobin variants. Int. J. Mach. Learn. Comput..

[50] Das R., Datta S., Kaviraj A., Sanyal S.N., Nielsen P., Nielsen I., Sharma P., Sanyal T., Dey K., Saha S. (2020). A decision support scheme for beta thalassemia and HbE carrier screening. J. Adv. Res..

[51] Srivastava P.C., Bevington J.M. (1973). Iron deficiency and-or thalassaemia trait. Lancet.

[52] Shine I., Lal S. (1977). A strategy to detect beta-thalassaemia minor. Lancet.

[53] Çil B., Ayyıldız H., Tuncer T. (2020). Discrimination of β-thalassemia and iron deficiency anemia through extreme learning machine and regularized extreme learning machine based decision support system. Med. Hypotheses.

[54] Purwar S., Tripathi R.K., Ranjan R., Saxena R. (2020). Detection of microcytic hypochromia using cbc and blood film features extracted from convolution neural network by different classifiers. Multimed. Tools Appl..

[55] St Pierre T., Aydinok Y., El-Beshlawy A., Bayraktaroglu S., Ibrahim A., Hamdy M., Pang W., Khorshid S., Bangma S., House M. (2022). P1505: Using Artificial Intelligence Neural Networks to Obtain Automated Liver Iron Concentration Measurements Using Magnetic Resonance Imaging—A Multi-Scanner Validation Study. Hemasphere.

[56] Positano V., Meloni A., Santarelli M.F., Pistoia L., Spasiano A., Cuccia L., Casini T., Gamberini M.R., Allò M., Bitti P.P. (2022). Deep Learning Staging of Liver Iron Content From Multiecho MR Images. J. Magn. Reson. Imaging.

